# Trade-Off between Redox Potential and the Strength of Electrochemical CO_2_ Capture in Quinones

**DOI:** 10.1021/acs.jpcc.2c03752

**Published:** 2022-08-12

**Authors:** Anna T. Bui, Niamh A. Hartley, Alex J. W. Thom, Alexander C. Forse

**Affiliations:** Yusuf Hamied Department of Chemistry, University of Cambridge, Lensfield Road, Cambridge CB2 1EW, United Kingdom

## Abstract

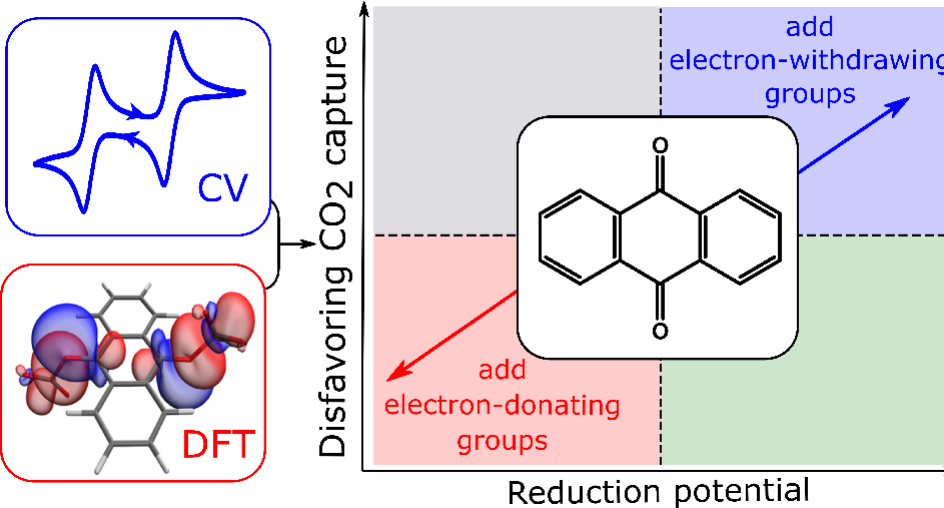

Electrochemical carbon dioxide capture recently emerged as a promising alternative approach to conventional energy-intensive carbon-capture methods. A common electrochemical capture approach is to employ redox-active molecules such as quinones. Upon electrochemical reduction, quinones become activated for the capture of CO_2_ through a chemical reaction. A key disadvantage of this method is the possibility of side-reactions with oxygen, which is present in almost all gas mixtures of interest for carbon capture. This issue can potentially be mitigated by fine-tuning redox potentials through the introduction of electron-withdrawing groups on the quinone ring. In this article, we investigate the thermodynamics of the electron transfer and chemical steps of CO_2_ capture in different quinone derivatives with a range of substituents. By combining density functional theory calculations and cyclic voltammetry experiments, we support a previously described trade-off between the redox potential and the strength of CO_2_ capture. We show that redox potentials can readily be tuned to more positive values to impart stability to oxygen, but significant decreases in CO_2_ binding free energies are observed as a consequence. Our calculations support this effect for a large series of anthraquinones and benzoquinones. Different trade-off relationships were observed for the two classes of molecules. These trade-offs must be taken into consideration in the design of improved redox-active molecules for electrochemical CO_2_ capture.

## Introduction

Anthropogenic carbon dioxide (CO_2_) emission is the major contributor to global climate change,^1^ which presents an urgent challenge to our society.^2,3^ One of the many important mitigation strategies for reducing greenhouse gas emissions is carbon capture. Conventional carbon capture technology involves wet chemical scrubbing using aqueous amines to absorb CO_2_ either at industrial sources or directly from the air for further sequestration.^4^ This strategy, however, has many limitations, including low capacities, poor oxidative stability, degradation in the presence of contaminants and large thermal energy demand for absorbent regeneration.^4−6^ In the search for new materials and technologies for carbon capture, electrochemically mediated CO_2_ capture has emerged as a promising approach.^7−10^ A popular strategy is to use redox-active organic compounds as CO_2_ carriers, with quinones being the most representative class of compounds.^11^ The electrochemical reduction of quinones generates oxyanion nucleophiles, which have high binding affinities toward CO_2_ and have been reported to concentrate and selectively separate CO_2_.^11−16^ The biggest advantage is that quinones can be electrochemically regenerated, leading to energy savings over traditional temperature- or pressure-swing processes.

While the recently reported use of quinone-functionalized electrodes is promising,^17^ questions remain about the molecular mechanism of this process and whether it is possible to increase its oxidative stability by carefully tuning the electron density of the quinones. In the presence of oxygen (O_2_), a loss in CO_2_ capture ability has been observed,^17^ as reduced quinones can be reoxidized while O_2_ is reduced.^18^ If the quinone structures can be tuned to have sufficiently positive reduction potentials by subsituting functional groups on the aromatic rings, it may be possible to avoid the side-reactions with O_2_. However, by tuning the redox potentials, one will also change the CO_2_ capture ability of the quinones. Early experimental work by DuBois first illustrated the trade-off between reduction potentials and CO_2_ binding constants.^11^ They found a linear relationship between the CO_2_ binding constant and the second reduction potential for a series of four functionalized benzoquinones and phenanthrenequinone. More recent experiments on benzoquinones have supported this trade-off,^19^ while other studies have further explored how functionalization impacts the chemistry of electrochemical CO_2_ capture by quinones.^20,21^

Despite this progress, a complete assessment of the relationship between redox potentials and CO_2_ capture abilities in a wide range of redox-active molecules has not been carried out. We believe this is because (i) quantifying the thermodynamic driving force of CO_2_ capture through CV experiments alone is challenging and (ii) a systematic electrochemical study of quinone derivatives with a different number of substituents and different functional groups is not easily accessible experimentally. Both these challenges can be tackled computationally through quantum chemical calculations. As quinones and their derivatives are also of interest in energy storage applications, several studies have computationally examined their redox properties, including the effect of electron-donating groups (EDGs) and electron-withdrawing groups (EWGs),^22,23^ substituent pattern effects,^24^ and structure–property relationships.^25^ Most of these works are based on density functional theory (DFT), which has proven to be an affordable and reliable way to study redox properties of organic molecules.^26,27^ However, it is worth emphasizing that these previous studies did not examine electrochemical CO_2_ capture.

In this article, we provide a detailed assessment of the relationship between redox potentials and CO_2_ capture abilities of different anthraquinone (AQ) and benzoquinone (BQ) derivatives. This is done both experimentally with CV and computationally with DFT calculations of the Gibbs free-energy change upon CO_2_ capture. Our results support a general trade-off between the redox potential and the strength of CO_2_ capture in a wide range of functionalized anthraquinones and benzoquinones. This trade-off has important implications in increasing the efficiency of future electrochemical CO_2_ capture technologies.

## Methods

### Experimental Section

#### Materials

Anthraquinone (AQ, 97%), octafluoroanthraquinone (AQ-F_8_, 96%), 1,4-methoxyanthraquinone (1,4-OMe-AQ, >99%), 1,4-difluoroanthraquinone (1,4-F-AQ, 98%), ferrocene (Fc), and tetrabutylammonium hexafluorophosphate (TBAPF_6_) were purchased from Sigma-Aldrich. 1-Chloroanthraquinone (1-Cl-AQ, 98%) was purchased from Thermo Fisher Scientific. 2-Chloroanthraquinone (2-Cl-AQ, >99%) was purchased from Chemcruz Enterprises Ltd. 1-Hydroxyanthraquinone (1-OH-AQ, >95%) was purchased from Cayman Chemical Company. Dimethyl sulfoxide (DMSO) was purchased from Thermo Fisher Scientific. All chemicals were used without further purification.

#### Electrochemical Experiments

Cyclic voltammetry measurements were carried out with a standard three-electrode cell using a BioLogic Sp-150 system with BioLogic EC-Lab software. Electrochemical measurements were conducted in a glass cell. The glassy carbon working electrode, the platinum wire counter electrode and the leak-free Ag/AgCl reference electrode were all purchased from Alvatek. After measurements were taken, 5 mM Fc was used as the internal standard. Tetrabutylammonium hexafluorophosphate in DMSO (0.1M) was used as the electrolyte. Before the gas was purged, 1 mM quinone was stirred into the electrolyte. The solution was purged under N_2_ for 2 h while stirring. For electrochemical tests with quinones under CO_2_, the electrolyte was bubbled with 100% CO_2_ for 2 h before experiments were run. All the measurements were recorded at a scan rate of 10 mV s^–1^.

### Computational Section

DFT calculations were performed in Q-Chem 5.3.^28^ Thermodynamic quantities were calculated for the species involved in the EEC and EECC mechanisms (detailed in the section Reaction Scheme and Oxyanion Nucleophilicity). For each quinone derivative involved, the gas-phase geometry was optimized at the B3LYP/6-311++G** level^29−32^ and the thermodynamic quantities were calculated at *T* = 298.15 K and *P* = 1 atm. For unsymmetrical quinone derivatives, species involved were modeled as isomers with minimal steric hindrance. Self-consistent field (SCF) calculations were considered to have converged when the wave function direct inversion in the iterative subspace (DIIS) error was less than 10^–11^. At the end of each successful geometry optimization, the structure was checked to ensure it was a real minimum on the potential energy surface and thermodynamic corrections were obtained by vibrational frequency analysis.

The SMD implicit solvation model^33^ has been reported to agree well with experimental data for the computation of reduction potentials of quinone derivatives.^22^ Using the optimized gas-phase geometries, single-point energy calculations were performed with the SMD model using dimethyl sulfoxide (DMSO) as the solvent. Gas-phase geometries were considered throughout to eliminate the dependency on the solvent model; this can be justified by the fact that there is almost no significant change in the geometry upon optimization in an implicit solvation model. Previously, it was also shown that for quinones, there is no real added value of performing geometry optimizations with implicit solvation, not to mention that these calculations are also computationally more demanding.^27^

The reduction potentials for the electron transfer steps were calculated by considering the half-reaction
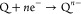
1For an *n*-electron reduction of quinones in solvent, the absolute reduction potential of the half reaction is given by the Nernst equation
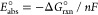
2where *G*_rxn_^°^ is the standard Gibbs free energy per coulomb of charge transferred during the reaction, *n* is the number of electrons, and *F* is the Faraday constant. The Gibbs free energy of a redox species in solution is given by
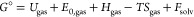
3where *U*_gas_ denotes the Born–Oppenheimer equilibrium potential energy, *E*_0,gas_ denotes the vibrational zero-point energy, *H* denotes the enthalpic contribution, *TS*_gas_ denotes the entropic contribution to the Gibbs free energy at *T* = 298.15 K in the gas phase, and *F*_solv_ denotes the solvation free energy from the solvation model. Ab initio calculations of reduction potentials performed using the thermodynamic cycle have been covered in more detail elsewhere.^34^ Since experimental reduction potentials are measured relative to a reference electrode potential, theoretical calculations are typically carried out for a half-cell reaction with the subtraction of a reference electrode. The ab initio *E*° might agree quantitatively with the experimental value; however, the two are not directly equivalent due to cell resistance, concentration dependence, or the lack of a better solvation treatment in calculations.^35,36^ Therefore, the potentials were calculated relative to those of a reference compound, rather than as absolute potentials, to remove the influence of systematic errors between the experimental conditions used here and those used to determine absolute potentials of reference compounds reported elsewhere. AQ was chosen as the reference compound, and isodesmic reaction schemes for the reduction and oxidation of are given as follows:
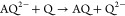
4Using the *ΔG*_rxn_^°^ = –*nFE*_rxn_^°^ relationship,
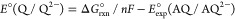
5For both EEC and EECC mechanisms, the electron transfer EE steps are considered a single two-electron reduction, and the calculated *E*° values are given in reference to Fc^+^/Fc. For the chemical steps C or CC, the standard Gibbs free energies (*ΔG*_C_^°^ or *ΔG*_CC_^°^) are given.

## Results and Discussion

### Reaction Scheme and Oxyanion Nucleophilicity

**Figure 1:**
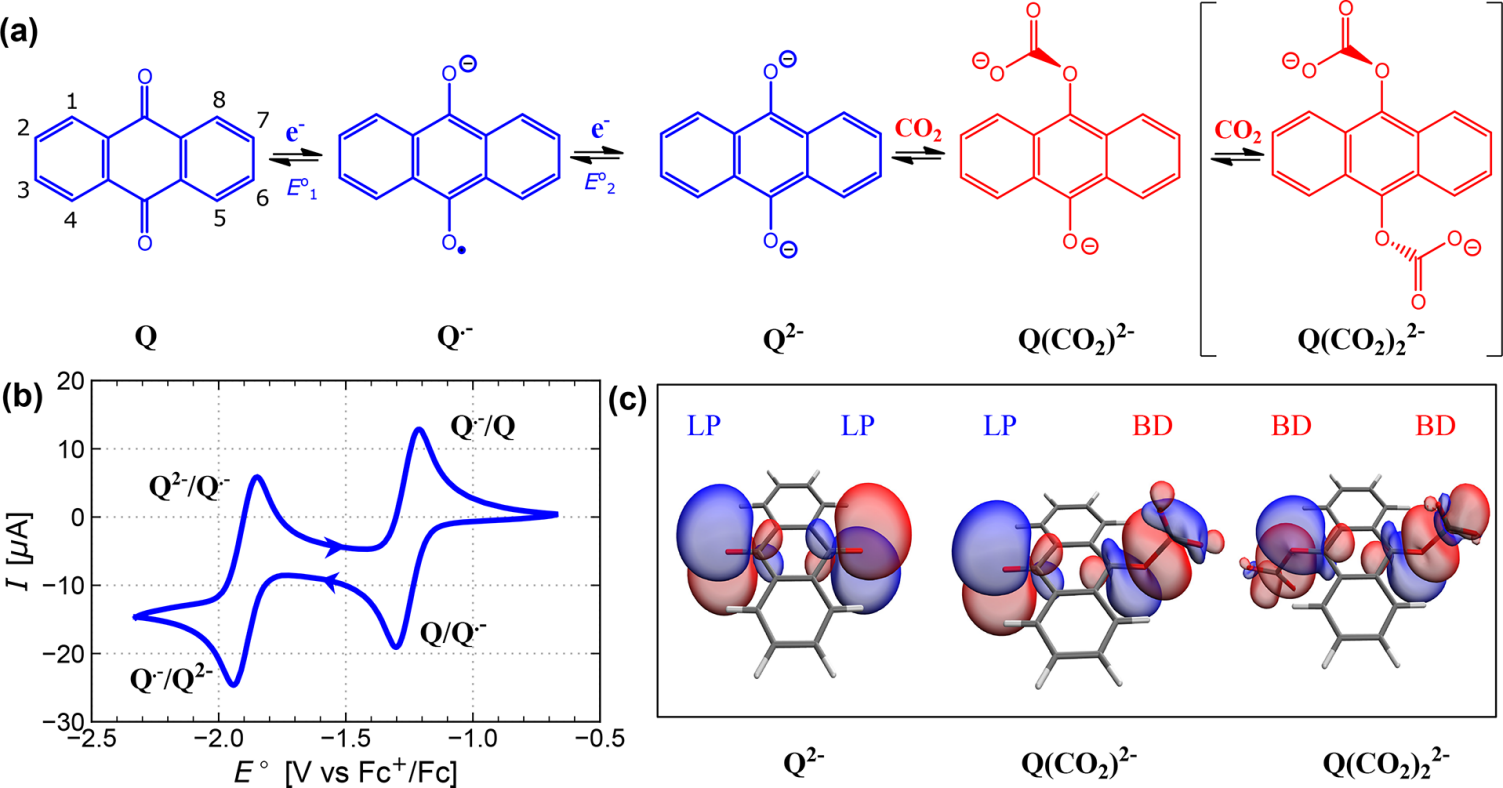
EEC and EECC reaction schemes for electrochemical CO_2_ capture by AQ. (a) The stepwise EEC reaction scheme employed in the DFT calculations includes two electron transfer steps followed by one chemical step. The EECC scheme includes one further chemical step. (b) Experimental CV of AQ (1 mM in DMSO) under N_2_ recorded at a scan rate of 10 mV s^–1^, with cathodic and anodic peaks labeled with oxidation and reduction species, respectively. (c) Orbitals representing the nucleophilic lone pairs (LP) and the O–CO_2_ bond (BD) formed based on the NBO analysis of DFT results.

In this work, we consider two different quinone-mediated electrochemical CO_2_ capture mechanisms, known as “EEC” and “EECC” (Figure 1a). In these mechanisms, two electron transfer (E) steps occur before any chemical (C) CO_2_ capturing step, which has been supported by a previous CV analysis of EWG-substituted quinones at a low CO_2_ concentration.^17,19−21,37^ Since our goal is to mitigate side-reactions of reduced quinones with O_2_, our main interest is to add EWGs to stabilize the reduced quinones, increasing the likelihood of both electrochemical reduction steps being required prior to any CO_2_ capture. The electrochemical reduction occurs as
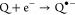
6
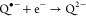
7where Q is the neutral quinone, Q^•–^ is the semiquinone radical anion, and Q^2–^ is the quinone dianion. In the absence of CO_2_, the CV of anthraquinone in DMSO exhibited the two expected reduction waves corresponding to the processes in eqs 6 and 7 at potentials of *E*_1_^°^ = −1.3 V and *E*_2_^°^ = −1.9 V vs Fc^+^/Fc, respectively, when midpoint potentials were used (Figure 1b). We further combine the two reduction steps into an “EE” step according to
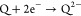
8since the combined two-electron reduction potential (*E*_EE_^°^) from DFT is also more accurate than the separate one-electron reduction potentials (*E*_1_^°^ and *E*_2_^°^), as detailed in the next section.

In the EEC scheme previously proposed as the capture mechanism for quinones with more than two EWG substituents,^11,19,20^ a single capture event occurs after electrochemical reduction.
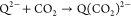
9Here Q(CO_2_)^2–^ is the monocarbonate dianion. The Gibbs free energy associated with this step is given as Δ*G*_C_^°^. We also investigate the EECC scheme proposed for quinones with mono- and disubstitutions of EWGs,^11,20^ where two capturing events are modeled as a “CC” step according to
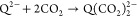
10Here Q(CO_2_)_2_^2–^ is the dicarbonate dianion, representing the theoretical maximum capture ability of quinones. The Gibbs free energy associated with this step is given as Δ*G*_CC_^°^.

Importantly, other electrochemical capture mechanisms are possible, most notably the ECEC scheme, where after the first reduction step Q^•–^ reacts with CO_2_ to form the semicarbonate radical anion Q(CO_2_)^•–^, as suggested in studies of EDG-substituted quinones at high CO_2_ concentrations.^12,16,21,37^ We do not consider this mechanism here due to the challenges in modeling CO_2_ capture by radical anions using DFT,^38−41^ which is a limitation of our study.

We first explored the CO_2_ capture steps for anthraquinone using DFT calculations. The localized orbital forms representing the lone pairs (LPs) and two-electron two-center bonds (BDs) were decomposed from the DFT wave function by natural bond orbital analysis (NBO) (Figure 1c).^42^ Upon reduction, the quinone C–O bond length increases (Table 1), and NBO analysis shows a change from the C–O π-orbital in Q to a lone pair on O in Q^2–^ (SI), which is consistent with the canonical Lewis structures. For each carbon capture event, a LP on an oxygen acts as a nucleophile and attacks a CO_2_ molecule, forming a C–O BD to CO_2_ (Figure 1c). It is clear that this oxygen LP and its electron density govern the nucleophilicity and CO_2_ capture ability of Q^2–^. Upon the second carbon capture event (which is only relevant in the EECC mechanism), the bond length of O–CO_2_ decreases and the quinone C–O bond length increases (Table 1), suggesting a decrease in bonding affinity toward CO_2_. This decrease is consistent with the carbonate group withdrawing electron density from the initially free oxyanion center.

**Table 1:** C–O Bond Lengths from DFT Calculations^a^

species	C–O quinone (Å)	O–CO_2_ (Å)
Q	1.220	
Q^•–^	1.257	
Q^2–^	1.291	
Q(CO_2_)^2–^	1.269/1.380	1.495
Q(CO_2_)_2_^2–^	1.356	1.553

a For Q(CO_2_)^2–^, the values are given for the C–O bond of the quinone on the sides where CO_2_ is and is not bound.

### Benchmarking Reduction Potentials of Substituted Anthraquinones from CV and DFT

**Figure 2:**
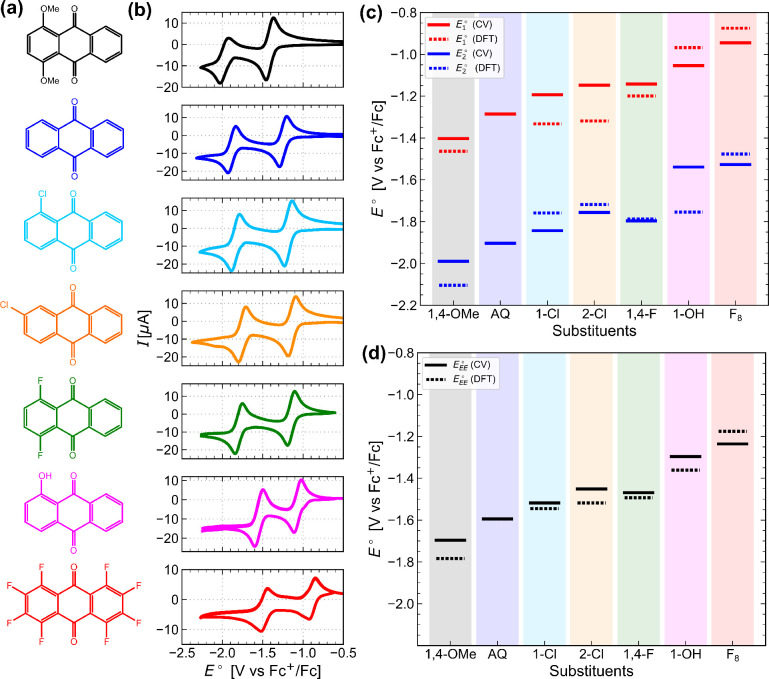
Reduction potentials of selected AQ derivatives. (a) Structures of all selected compounds. (b) CVs of AQ derivatives (1 mM in DMSO) recorded at a scan rate of 10 mV s^–1^ under N_2_. (c) Comparison of single-reduction potentials from CV and DFT. (d) Comparison of double-reduction potentials from CV and DFT. DFT reduction potentials were calculated with reference to AQ.

Before proceeding to study the effect of quinone functionalization on electrochemical CO_2_ capture, we compared the computed and experimental CV redox potentials for selected AQ derivatives to evaluate the reliability of the DFT calculations. Figure 2a shows structures of the derivatives and Figure 2b shows the corresponding CVs in DMSO under N_2_. The midpoint potentials of the pair of redox peaks in the CV were taken as single-reduction potentials *E*_1_^°^ and *E*_2_^°^. The average of *E*_1_^°^ and *E*_2_^°^ was then taken as the double-reduction potential *E*_EE_^°^. The corresponding values calculated from DFT (detailed in the Methods section) are compared to experimental single and double reduction potentials, as shown in Figure 2c and d, respectively.

In all cases, *E*_1_^°^ is more positive than *E*_2_^°^, as it is easier to reduce Q than Q^•–^. This is because (i) the gain of an electron is more favorable for neutral Q than for negatively charged Q^•–^ on electrostatic grounds and (ii) there is a greater contribution from solvation to the reduction driving force for the second reduction than the first reduction (Figure S4).

When EWGs are substituted, as in the case of -Cl and -F, the reduction potentials become more positive for both CV and DFT results (Figure 2c and d, respectively). This trend agrees with previous studies^21,22,25^ and is consistent with the electron density on the reduced quinones being more delocalized, making reduction more thermodynamically favorable. Positional effects observed in the experiments and calculations for the case of -Cl also agree; substitution at the 2-position shifts *E*_EE_^°^ to a more positive value than that at the 1-position. This trend is consistent with greater delocalization of electron density when Cl is substituted at the 2-position with a larger LUMO coefficient in Q (Figure S1). The largest shift of 0.4 V due to multiple substitutions in the case of octafluoroanthraquinone AQ-F_8_ is also well predicted by DFT.

When EDGs are substituted, as in the case of -OMe, the reduction potentials become more negative for both the CV and DFT results as the reduced forms become disfavored. In the case of -OH, however, intramolecular hydrogen-bonding stabilizes the Q^2–^ form such that an overall increase in *E*_EE_^°^ is observed. This increase is supported by the change in the DFT geometry, where the hydroxyl proton in Q is transferred to the quinone O following electrochemical reduction (Figure S2).

While the computed *E*_EE_^°^ values generally compare favorably with experimental measurements, with errors less than 0.05 V, there are greater deviations in the trend for *E*_1_^°^ and *E*_2_^°^. We believe this due to a well-known problem in DFT of describing certain radical anions of atoms and small molecules.^38−41^ Treating EE as a single two-electron reduction step (eq 8) allows the radical semiquinone anion Q^•–^ to be bypassed, the accurate treatment of which calls for a higher level of theory. Overall, the good agreement between computed and experimental *E*_EE_^°^ values validated the use of the DFT approach to study the electrochemical reduction of quinones and enabled us to explore a wider range of functional group substitutions, as shown in the next section.

### Effect of Tuning the Quinone Electron Density on the Electrochemical CO_2_ Capture Thermodynamics

We now explore how varying the electron density of quinones impacts electrochemical CO_2_ capture through a F-substituted AQ series. F is a good candidate to use for fine-tuning because (i) F substituents tend to show a strong negative inductive effect of an EWG that predominates the positive resonance effect of an EDG,^24^ (ii) there are various ways to fluorinate AQ selectively,^43−45^ and (iii) the small size of F minimizes steric effects, whereas larger substituents can decrease the CO_2_ capture ability, as previously observed in Cl-substituted quinones.^20^

**Figure 3:**
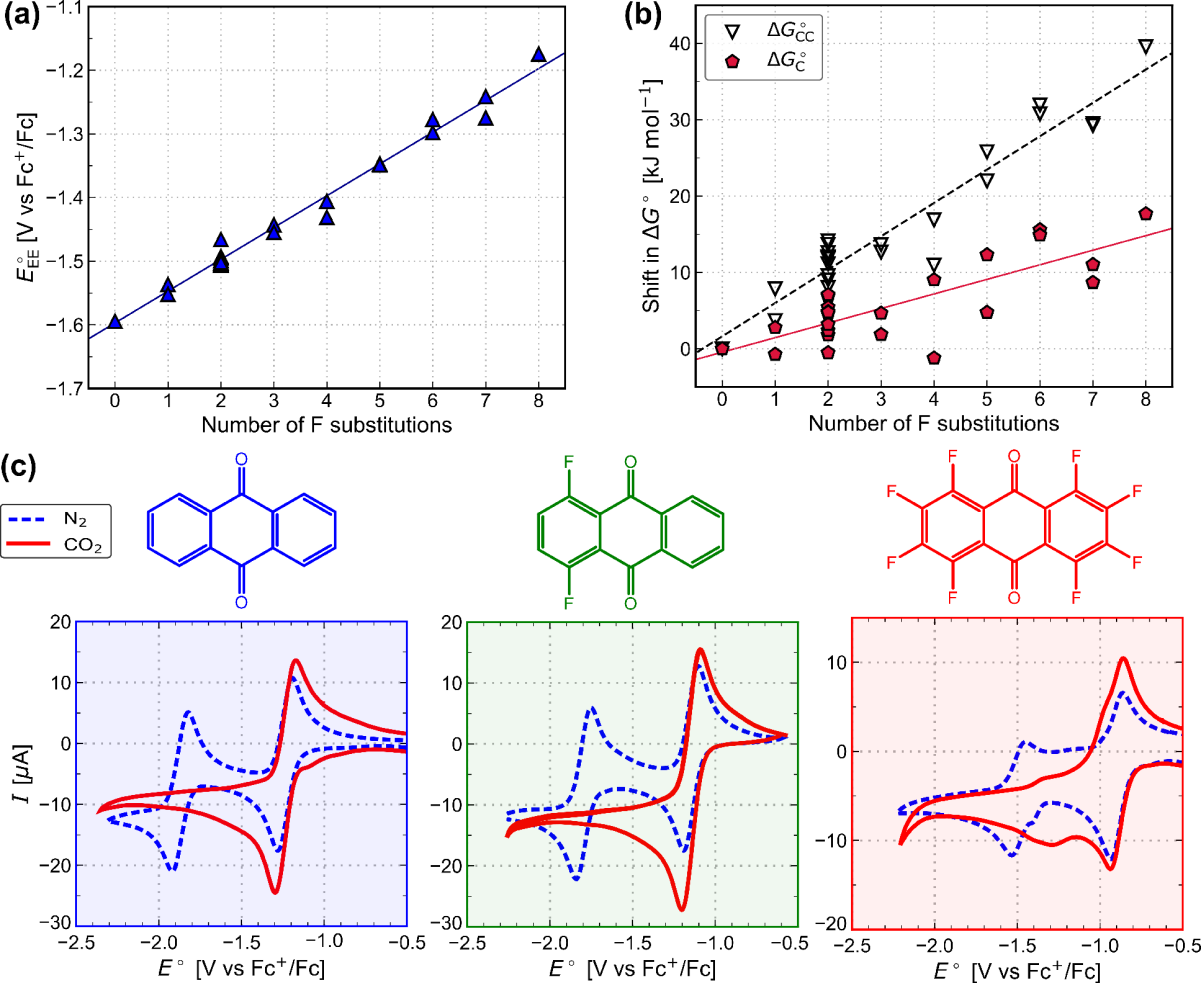
Calculated reduction potentials and capture abilities of F-substituted AQ derivatives. (a) Variation in the reduction potential for two-electron reduction. (b) Variation in the Gibbs free-energy changes for single CO_2_ capture (EEC scheme) and double CO_2_ capture (EECC scheme). The shifts are plotted relative to unsubstituted AQ. The actual energies and positions of the substutions are given in the SI. (c) Experimental CVs of unsubstituted AQ, 1,4-F-AQ, and AQ-F_8_ recorded at a scan rate of 10 mV s^–1^ under both N_2_ and CO_2_.

The thermodynamics governing the electron transfer and chemical steps for AQ derivatives with an increasing number of F substituents were quantified using DFT calculations. Figure 3a shows that the computed *E*_EE_^°^ values increase from −1.54 to −1.17 V against Fc^+^/Fc upon the substitution of 1–8 F atoms, respectively, and is generally directly proportional to the number of F substitutions made. As more electron density is withdrawn from the oxygens in the Q^2–^ anion, the reduced form is more delocalized and stabilized against reoxidation. The increase in the reduction potential varies slightly for different substitution positions for F, as seen previously in the cases of other substituents.^22−24^ When EDGs are substituted instead, the opposite effect is observed (Figure S3). Overall, increasing the number of F substitutions leads to an approximately linear increase in the reduction potentials of AQ. In the context of electrochemical CO_2_ capture, the EE step (eq 8) is more thermodynamically favorable, as the electron density of quinones is tuned by the increasing number of EWGs, which should impart improved stability in oxygen.

We then evaluated the thermodynamics of the chemical CO_2_ capture step for our F-substituted AQ series (Figure 3b). For the EEC scheme, the computed Gibbs free energy change *Δ*G_C_^°^ increases by 20 kJ mol^–1^ upon the substitution of 1–8 F atoms. Again, the effect is approximately linear (solid line in Figure 3b), and there is no consistent trend in the position of substitution. This effect can be explained by considering the electron density available in Q^2–^ for bonding with CO_2_ to form Q(CO_2_)^2–^. AQ-F_8_ has a lower charge density on the oxygen than unsubstituted AQ in its Q^2–^ form and therefore has a lower affinity for binding to CO_2_. The O–CO_2_ bond length of the Q(CO_2_)^2–^ form calculated by DFT is 1.529 Å in AQ-F_8_ compared to 1.495 Å in AQ, indicating that a weaker bond is formed due to more electron density being delocalized away from the oxygens in Q^2–^. Similarly for the EECC scheme, increasing the number of F substitutions also leads to an approximately linear increase in the Gibbs free-energy change Δ*G*_C_^°^ in the capture of two CO_2_ (dashed line in Figure 3b). The trend line for the EECC scheme has a gradient slightly larger than twice that of the EECC scheme, which consistent with the second CO_2_ being less thermodynamically favorable than the first (Figure S4). Overall, in the context of electrochemical CO_2_ capture, the chemical steps become less thermodynamically favorable as more EWGs are added, supporting a trade-off between redox potential and CO_2_ affinity, as suggested by previous experimental work on functionalized benzoquinones.^11,19^

To explore this result experimentally, we recorded the CVs of unsubstituted AQ, 1,4-F-AQ, and AQ-F_8_ under both N_2_ and CO_2_ (Figure 3c). For all cases, the reduction wave positions of the first electron transfer (eq 6) remain unchanged in the presence and absence of CO_2_. This behavior is in line with the quinone in all cases needing to be first activated by reduction to Q^•–^ or Q^2–^ before capturing CO_2_, as observed in previous studies.^21^ For both AQ and 1,4-F-AQ, the second reduction wave (eq 7) appears to shift underneath the first reduction wave, in agreement with previous work.^16,17^ In contrast, for AQ-F_8_, the shift of the second reduction wave is smaller, and two redox processes can be clearly observed under CO_2_. The smaller positive shift of the second reduction peak the indicates that the stabilization of the reduced species by CO_2_ is lower for AQ-F_8_ than for AQ and 1,4-F-AQ and suggests a smaller binding constant to CO_2_,^19,21^ as predicted by our DFT calculations. The reduction in the height of the second reduction wave for AQ-F_8_ is an indication of some reactivity with CO_2_, forming either Q(CO2)^2–^ or Q(CO_2_)_2_^2–^ species.^12^ Overall, these experimental results are qualitatively consistent with the predictions of our DFT calculations, providing support for the calculated trade-off between the redox potential and the CO_2_ capture strength. A more detailed study of the detailed capture mechanisms of these molecules is beyond the scope of this work and is under investigation in our laboratory.

### Trade-Off between the Redox Potential and the Strength of CO_2_ Capture

**Figure 4:**
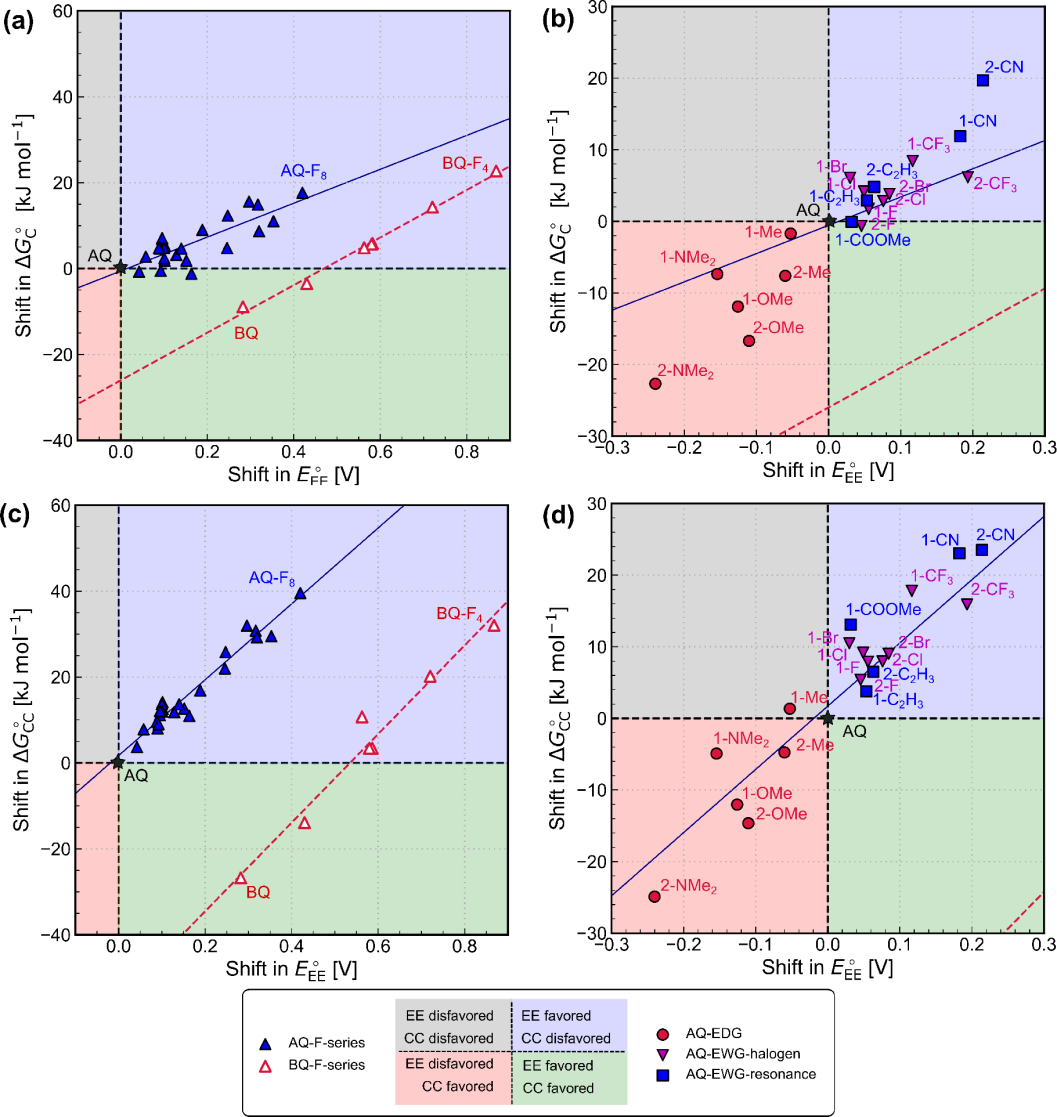
Trade-off between the redox potential and the strength of CO_2_ capture for the (a and b) EEC and (c and d) EECC mechanisms. The computed shift in the Gibbs free-energy change for the chemical steps is plotted against the shift in the two-electron reduction potential EE in DMSO computed for (a and c) multiple F-substituted AQ and BQ derivatives and (b and d) the monosubsituted AQ derivatives. The solid blue line and the dashed red line are the best fits for the F-series of AQ and BQ, respectively. The shifts are plotted relative to unsubstituted AQ.

The above results indicate that by adding EWGs we are simultaneously making electrochemical reduction more favorable and carbon capture less favorable, supporting the early work of DuBois.^11^ Therefore, when designing quinone derivatives for electrochemical CO_2_ capture, to enhance efficiency, a balance between favoring electron transfer and chemical steps must be taken into account. Focusing on the EEC scheme of the F-substituted AQ series, this trade-off can be seen clearly by combining panels a and b in Figure 3 into a plot of the shift in Δ*G*_C_^°^ versus the shift in *E*_EE_^°^, as shown in Figure 4a. The shifts are plotted relative to the unsubstituted AQ and regions, where each step is favored or disfavored and color-coded accordingly. The linear solid blue trend line that emerges confirms the unfavorable relationship between the thermodynamics of cathodic activation and the nucleophilic addition of CO_2_.

An avenue to break the observed trade-off could be to explore alternative capture agents beyond anthraquinone. Calculations on substituted benzoquinones (BQ) also revealed a trade-off between the redox potential and the CO_2_ capture strength (dashed red line in Figure 4a), though interestingly we found that both the reduction and carbon capture steps become more thermodynamically favorable relative to AQ, i.e., different trade-off relationships were found for functionalized BQ and AQ molecules. The downside of BQ, however, is that its fine-tuning ability is limited by the total number of possible substitutions. BQs are also more electrochemically unstable and susceptible to side reactions and degradation.^46^ Therefore, other redox-active molecules, such as naphthoquinones, might be able to provide a better compromise and are worth investigating in future work.

After demonstrating this trade-off for the F-substituted AQ and BQ series, we posed the question of whether it was possible to overcome this trade-off using other functional groups, i.e., can we favor the EE step without disfavoring the C step significantly and thus falling below the linear fit (blue solid lines in Figure 4a and b). We therefore explored a range of monosubstituted AQ derivatives with different functional groups (Figure 4b), including -F, -Cl, -Br, and -CF_3_ as halogen-based EWGs; -C_2_H_3_ (ethylene), -COOMe, and -CN as resonance-based EWGs; and -CH_3_, -OMe, and -NMe_2_ as EDGs. Indices 1 and 2 indicate the positions of substitution,^48^ and the plot is again color-coded according to how the thermodynamics of cathodic activation and nucleophilic addition differ from those of unsubstituted AQ. The unfavorable relationship still remains: the substitution of EWGs favors the EE step but disfavors the C step, while the substitution of EDGs favors the C steps but disfavors the EE steps. For most substituents, substitution closer to the carbonyl group at position 1 leads to a higher Δ*G*_C_^°^ due to steric hindrance between the captured CO_2_ and the substituents. The tuning window of these single substitutions is approximately 0.5 V for *E*_EE_^°^ and 40 kJ mol^–1^ for Δ*G*_C_^°^. Although these monosubstituted derivatives do not all fall on the same linear fit for the F-substituted AQ series, the deviation from the trend is small. It is useful to tie these results back to the role of the electron density in the oxyanion Q^2–^. Delocalization of the electron density around the ring would stabilize the quinone in its dianion Q^2–^ form, meaning the quinone is less likely to be reoxidized by O_2_ after being electrochemically reduced but is also less likely to have enough nucleophilicity to react with CO_2_.

For the EECC scheme, the shift in Δ*G*_CC_^°^ is plotted against the shift in *E*_EE_^°^ for the F-substituted AQ series (Figure 4c) and a range of monosubstituted AQ derivatives (Figure 4d). A similar trade-off between the thermodynamics of cathodic activation and the nucleophilic addition of CO_2_ is seen for the EECC scheme, which has a larger gradient than that for the EEC scheme. It would therefore still be very difficult to fine-tune the electron density of AQ using these functional groups to favor both the electron transfer and chemical steps.

Overall, we have demonstrated that the trade-off between the redox potential and the strength of electrochemical CO_2_ capture is general to the fine-tuning of quinones by functional group substitutions through (i) a different number of substituents and (ii) different types of substituents. We have also demonstrated that different trade-offs exist for different types of quinones (AQ versus BQ) and that the trade-offs are general to both the EEC and EECC mechanisms. This raises questions about whether functional group substitutions can be used to address oxygen sensitivity issues in these systems while maintaining sufficient CO_2_ reactivity.

To assess the impact of oxygen, the CV of unsubstituted AQ under O_2_ in DMSO was recorded. The changes in the data support some reactivity of AQ with O_2_ (Figure S5). To avoid the possibility of parasitic reactions after cathodic activation, intuitively one would select a quinone with EWGs and a more positive reduction potential, assuming that the reduction potential is both a measure of how likely the reduced quinone becomes reoxidized by oxygen or chemically reacts with an electrochemically reduced oxygen species. However, as we have shown through DFT, the reactivity of the quinone to CO_2_ is weaker (main implication of the trade-off). Through CV experiments, we also showed the change in the form of the CV, with a smaller positive shift of the second reduction peak. Therefore, just naively adding EWGs not only reduces the CO_2_ capture ability but also does not necessarily solve the O_2_ reactivity problem. To this end, we provide some rough guidelines for fine-tuning to optimize the efficiency of quinone-mediated electrochemical CO_2_ capture in DMSO:

(i) Unsubstituted anthraquinone shows some reactivity with O_2_, so some sort of substitution is likely to be necessary for this molecule.
(ii) Substitutions of bulky functional groups are undesirable on steric grounds.
(iii) For anthraquinone, substitutions at the 2-position create less steric hindrance for CO_2_ capture than those at the 1-position and thus are advantageous for tuning the redox potentials while maintaining CO_2_ reactivity.
(iv) A combination of different functional groups for multiple substitutions might be necessary, i.e., EDGs at certain positions and EWGs at others, to avoid the trade-off.
(v) Functionalized anthraquinones and benzoquinones are subject to different trade-off lines, so the exploration of different classes of molecules is a promising avenue in the search for oxygen-stable electrochemical CO_2_ capture.

These guidelines are admittedly crude, relying on the assumptions that the reaction with CO_2_ proceeds through an EEC or EECC mechanism and that the half-wave potential of O_2_ does not change in the presence of quinone species. Moreover, other factors, including but not limited to CO_2_ concentration, temperature, electrolyte concentrations, and solvents, are likely to affect the desired properties of the quinones. The incorporation of these factors goes beyond the scope of the present article and these guidelines and therefore require full validation by explicit experiments and calculations in the presence of O_2_. However, the finding of a general trade-off between the redox potential and the strength of CO_2_ capture is of value for the design of improved redox active molecules for electrochemical carbon dioxide capture.

## Conclusions and Outlook

In this article, we have investigated the effect of functional group substitution on the thermodynamics of electrochemical CO_2_ capture by quinones. Using the EEC and EECC schemes to describe the overall capture process, we have identified the electron density of the oxyanion Q^2–^ species as the key factor governing the thermodynamics of the electrochemical reduction and carbon capture steps. After benchmarking DFT calculations of the electrode potentials relative to experimental values, we quantified the thermodynamic driving force of the electrochemical reduction and carbon capture steps.

Our key findings are as follows:

(i) There is a general trade-off between redox potentials and the strength of CO_2_ capture in a wide range of functionalized anthraquinones for the EEC and EECC mechanisms studied.
(ii) There is a need to fine-tune the quinones such that the electrochemical reduction steps are favored (so side-reactions with O_2_ are disfavored) without disfavoring the carbon capture steps significantly.
(iii) The steric bulk of the functional groups and their positions relative to the quinone oxygen impact the calculated CO_2_ reactivity.
(iv) Different types of redox-active molecules are subject to different trade-off relationships (e.g., benzoquinoes vs anthraquinones), so the exploration of new classes of molecules may lead to oxygen-stable electrochemical CO_2_ capture in the future.
(v) Different capture mechanisms (e.g., EEC vs EECC) are subject to trade-off relationships with different magnitudes (gradients).

While we focused on anthraquinone and benzoquinone derivatives in DMSO, the methods employed here can be readily extended to other redox-active molecules, including other types of quinones^14,15^ and sulfides.^47^ Effects of other aprotic solvents can be easily extended with the current method using implicit solvation models, while those of ionic liquids in recent developments^15,16^ are likely to call for combination with more expensive hybrid quantum mechanics/molecular mechanics (QM/MM) approaches.

The trade-off between the redox potential and the strength of CO_2_ capture has important implications in electrochemical carbon capture processes. As the unfavorable relationship comes from the inherent role of the electron density on the nucleophile, we hope to motivate the development of novel ways to work around this trade-off in the rational design of new CO_2_ capture materials. Of particular relevance to this work is the recent study by Simeon et al.,^21^ which suggested that quinones with EDG substitutions are more suitable candidates for capturing CO_2_ without side reactions with O_2_. It was proposed that these undergo a ECEC scheme, which was attributed to the disappearance of the second reduction wave in their CVs. Combining these CV analyses with the approach we employ of using DFT, one can investigate the thermodynamics of different potential capture mechanisms and see if such a trade-off still persists. We also note that very recent work has shown that electrolyte additives such as alcohols offer powerful means of tuning electrochemical CO_2_ capture thermodynamics^19^ and can potentially overcome trade-offs between the redox potential and the carbon capture strength. Overall, our work supports a general trade-off between the redox potential and the strength of electrochemical CO_2_ capture and provides a foundation for the design of improved molecules and materials that can mitigate greenhouse gas emissions.

## Notes

The data that support the findings of this study, including optimized geometries of compounds and DFT energy calculations, are openly available at the University of Cambridge Data Repository at 10.17863/CAM.85559.

## Supporting Information Available

Orbital analysis of species in EEC and EECC, hydrogen bonding in the OH case, substitutions of the Me series, movement from the gas phase to the solution phase, DFT reduction potentials and free energy changes, and the CV of AQ under O_2_ (PDF)
