# Supplementary material for: Trade-Off between Redox Potential and the Strength of Electrochemical CO2 Capture in Quinones
Source: J Phys Chem C Nanomater Interfaces. 2022 Aug 12;126(33):14163–72. doi: 10.1021/acs.jpcc.2c03752 (PMC9421892; doi:10.1021/acs.jpcc.2c03752)
Supplement: Supplementary file 1 — jp2c03752_si_001.pdf [file jp2c03752_si_001.pdf]

Supporting Information: Trade-off between Redox Potential and Strength of Electrochemical CO_2 Capture in Quinones

Anna T. Bui, Niamh A. Hartley, Alex J. W. Thom,* and Alexander C. Forse*

Yusuf Hamied Department of Chemistry, University of Cambridge, Lensfield Road, Cambridge, CB2 1EW, United Kingdom

E-mail: ajwt3@cam.ac.uk; acf50@cam.ac.uk

Contents

1 Orbital analysis of species in EEC and EECC **S-2**

2 Hydrogen bonding in OH case **S-4**

3 Substitutions of Me-series **S-5**

4 Going from gas phase to solution phase **S-6**

5 DFT reduction potentials and free energy changes **S-7**

6 CV of AQ under O_2 **S-9**

References **S-10**

1 Orbital analysis of species in EEC and EECC

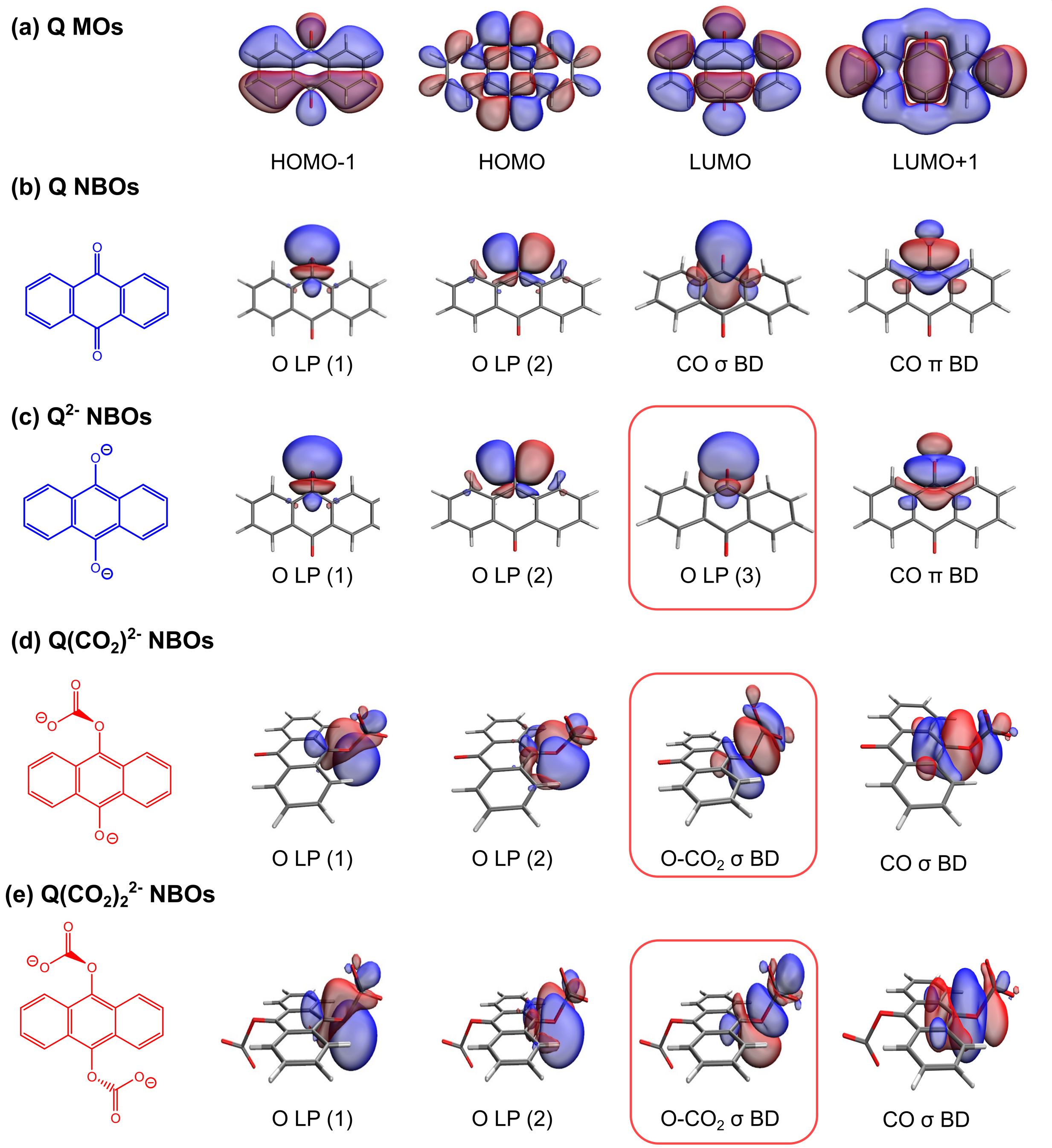

Figure S1: Orbital analysis from DFT wavefunction for species involved in the EEC and EECC mechanisms. (a) The frontier molecular orbitals (MOs) of anthraquinone in unreduced state (Q). (b) The natural bonding orbitals (NBOs) of the unreduced state (Q), (c) the reduced form Q^{2-}, (d) the monocarbonate $Q(CO_2)^{2-}$ and (e) the dicarbonate $Q(CO_2)_2^{2-}$. The boxed NBOs are directly involved in the capture of CO_2.

The frontier molecular orbitals (FMO) of the neutral Q are shown in Figure S1(a). The singly occupied molecular orbital (SOMO) of $Q^{\bullet -}$ and the HOMO of Q^{2-} have the same form as the LUMO of Q, which is consistent with electrons being added to the LUMO upon reduction. The LUMO energy has been shown to correlate with the reduction potential.[S1] The coefficient of the LUMO of Q on the 2- position is larger than the 1- position so in Q^{2-}, more electron density lies on the 2- position than the 1- position and this has implication on effect of position of substitution. As discussed in the main text, there is greater delocalisation of electron density when an EWG is substituted at the 2- position, resulting in E°_{EE} being more positive than a substitution at the 1- position.

Natural bonding orbital (NBO) analysis[S2] is carried out to optimally transform the DFT wavefunction into localised forms, corresponding to the one-centre lone pair (LP) and two-centre bond (BD) elements. The NBOs involving the O centre which captures CO_2 for each species in the EECC scheme are shown in Figure S1(b-e).

2 Hydrogen bonding in OH case

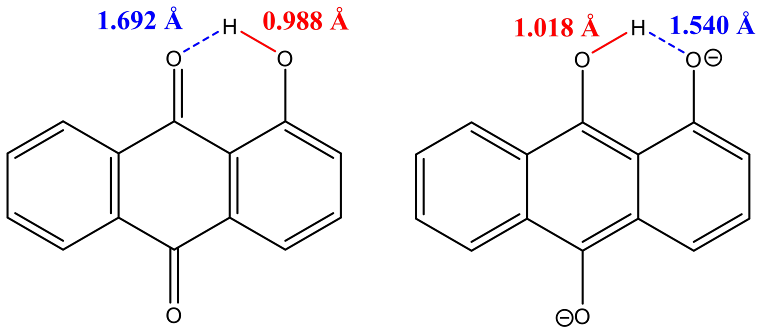

Q **Q2-**

Figure S2: Effect of intramolecular hydrogen bonding on the OH bond lengths of the neutral and reduced form of 1-OH-AQ from DFT

When EDGs are substituted, the reduction potentials are expected to become more negative as the reduced forms become disfavoured. In the case of -OH, however, intramolecular hydrogen-bonding stabilises the Q^{2-} form so an overall increase in E°_{EE} is observed. This is supported by the change in DFT geometry: the hydroxyl proton in Q transfers to quinone O following electrochemical reduction, as depicted by the change in OH bond lengths, as shown in .

3 Substitutions of Me-series

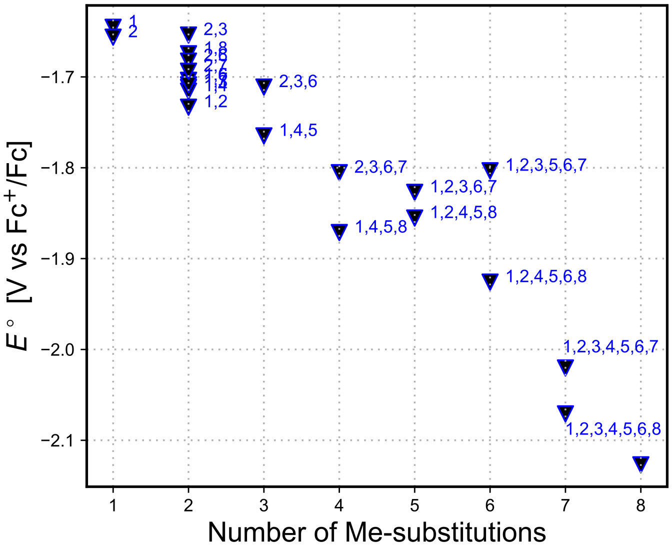

Figure S3: Variation in the reduction potential for two-electron reduction for Me-substituted AQ derivatives

Figure S3 shows that the computed E°_{EE} decreases from -1.63 V to -2.13 V against Fc^{+}/Fc upon substitution of one to eight Me, and is generally inversely proportional to the number of Me substitutions made. As more electron density is withdrawn from the Os in the Q^{2-} anion, the reduced form is more delocalised and stabilised against re-oxidation. The increase in reduction potential varies slightly for different positions of substitutions for Me, as seen previously in the case of F substitutions. Overall, increasing the number of EDGs leads to approximately linear decrease in the reduction potentials of AQ, which is the opposite effect by F substitutions shown in the main text.

4 Going from gas phase to solution phase

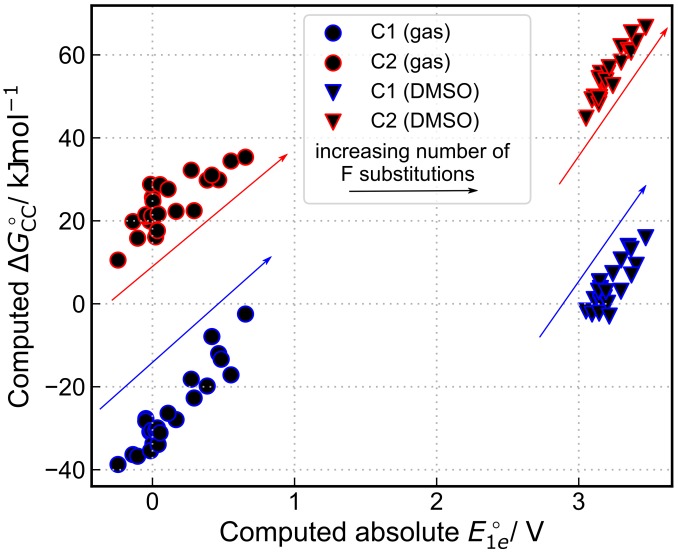

Figure S4: Computed Gibbs free energy change and redox potentials for the F-series in the gas and solvated phases.

For a more detailed look at how thermodynamic quantities are changed going from gas to solution phase, the Gibbs free energy change for each chemical step C1 and C2 (Eqs (3,4) in main text) is plotted against the absolute 2-electron reduction potential (Eq (5) in main text) computed for the F-subsituted AQ derivatives in both gas phase and in DMSO, as shown in Figure S4. For each F-series plotted, there is a linear relationship between the reduction potential and the Gibbs free energy change of both chemical steps. In both gas phase and in DMSO, there is a larger Gibbs free energy change and therefore a smaller driving force for the capture of the second CO_2 than the first CO_2. This is supported by with the O$-$$CO_2$ bond lengths increasing from 1.495 Å to 1.553 Å going from $Q(CO_2)^{2-}$ to $Q(CO_2)_2^{2-}$ for AQ. Solvation by the SMD model increases the reduction potentials of all derivatives, accounting for the gain in solvation energy going from the neutral Q to the anion Q^{2-}. This gain in solvation energy is larger for AQ than for AQ-F_8 as AQ-F_8 in its neutral form is already well solvated. Therefore, the redox window in the solution phase is narrower than that in the gas phase.

5 DFT reduction potentials and free energy changes

The reduction potentials and free energy changes associated with the EEC and EECC schemes from DFT calculations (B3LYP/6-311++G** level,[S3–S6] SMD solvation model with DMSO[S7]) are given in Tables S1, S2 and S3.

Table S1: Reduction potential of EE step and free energy changes of C step and CC step for F-substituted AQ series from DFT calculations.

Species	E°_{EE} [V against Fc^+/Fc]	ΔG°_{C} [kJ mol^{-1}]	ΔG°_{CC} [kJ mol^{-1}]
AQ	-1.59	-1.75	43.0
AQ−F (1)	-1.54	1.02	51.9
AQ−F (2)	-1.55	-2.48	46.7
AQ−F_2 (1,2)	-1.50	5.14	54.1
AQ−F_2 (1,3)	-1.49	0.07	55.6
AQ−F_2 (1,4)	-1.49	3.70	57.2
AQ−F_2 (1,5)	-1.49	3.09	56.7
AQ−F_2 (1,6)	-1.49	0.65	55.0
AQ−F_2 (1,7)	-1.50	5.33	55.1
AQ−F_2 (1,8)	-1.47	1.45	54.8
AQ−F_2 (2,3)	-1.50	2.89	51.0
AQ−F_2 (2,6)	-1.51	3.04	52.6
AQ−F_2 (2,7)	-1.50	-2.26	52.0
AQ−F_3 (1,4,5)	-1.44	0.12	55.7
AQ−F_3 (2,3,6)	-1.46	2.90	56.7
AQ−F_4 (1,4,5,8)	-1.43	-2.97	54.0
AQ−F_4 (2,3,6,7)	-1.41	7.28	59.9
AQ−F_5 (1,2,3,6,7)	-1.35	10.6	68.8
AQ−F_5 (1,2,4,5,8)	-1.35	3.02	65.0
AQ−F_6 (1,2,3,5,6,7)	-1.30	13.8	74.9
AQ−F_6 (1,2,4,5,6,8)	-1.28	13.1	73.8
AQ−F_7 (1,2,3,4,5,6,7)	-1.24	9.28	72.5
AQ−F_7 (1,2,3,4,5,6,8)	-1.28	6.94	72.3
AQ−F_8	-1.17	15.9	82.6

Table S2: Reduction potential of EE step and free energy changes of C step and CC step for F-substituted BQ series from DFT calculations.

Species	E°_{EE} [V against Fc^{+}/Fc]	$\Delta G^{\circ}_{\mathrm{C}}$ [kJ mol^{-1}]	$\Delta G^{\circ}_{\mathrm{CC}}$ [kJ mol^{-1}]
BQ	-1.31	-10.6	16.3
BQ−F	-1.17	5.25	29.1
BQ−F_2 (m)	-1.01	4.10	46.4
BQ−F_2 (o)	-1.02	3.93	46.4
BQ−F_2 (p)	-1.03	3.09	53.7
BQ−F_3	-0.87	12.5	63.2
BQ−F_4	-0.73	20.9	75.0

Table S3: Reduction potential of EE step and free energy changes of C step and CC step for mono-substituted AQ series from DFT calculations.

Species	E°_{EE} [V against Fc^{+}/Fc]	$\Delta G^{\circ}_{\mathrm{C}}$ [kJ mol^{-1}]	$\Delta G^{\circ}_{\mathrm{CC}}$ [kJ mol^{-1}]
AQ−NMe_2 (1)	-1.75	-9.11	38.1
AQ−NMe_2 (2)	-1.84	-24.4	18.1
AQ−OMe (1)	-1.72	-13.7	31.0
AQ−OMe (2)	-1.71	-18.4	28.4
AQ−Me (1)	-1.65	-3.46	44.4
AQ−Me (2)	-1.66	-9.36	38.2
AQ−F (1)	-1.54	0.00	50.9
AQ−F (2)	-1.55	-2.39	48.4
AQ−Br (1)	-1.57	4.33	53.5
AQ−Br (2)	-1.51	2.08	52.0
AQ−Cl (1)	-1.55	2.43	52.2
AQ−Cl (2)	-1.52	1.09	51.0
AQ−CF_3 (1)	-1.48	6.69	60.8
AQ−CF_3 (2)	-1.40	4.42	58.9
AQ−C_2H_3 (1)	-1.54	1.17	46.8
AQ−C_2H_3 (2)	-1.53	3.06	49.5
AQ−COOMe (1)	-1.56	-1.82	56.1
AQ−CN (1)	-1.41	10.1	66.1
AQ−CN (2)	-1.38	17.9	66.5

6 CV of AQ under O_2

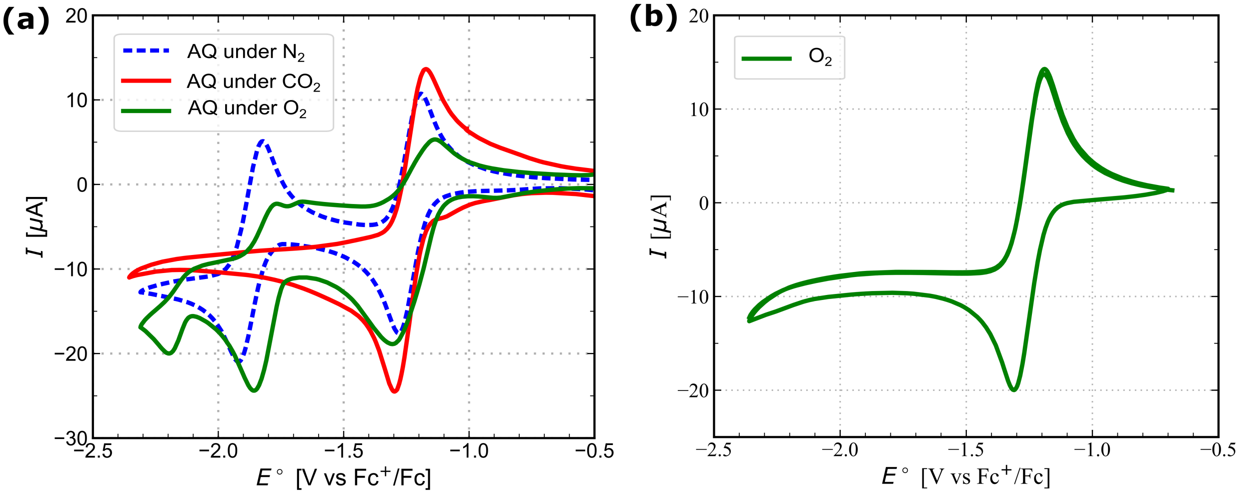

Figure S5: (a) CVs of unsubstituted AQ under O_2, CO_2, N_2 in DMSO at a scan rate of $10\,mV\,s^{-1}$. (b) CV of O_2 in DMSO at a scan rate of $10\,mV\,s^{-1}$.

The CV of unsubstituted AQ under O_2 in DMSO shows weaker oxidation waves compared to the case under N_2 and under CO_2. This indicates the reactivity of unsubstituted AQ with O_2.

Figure S5 shows the CV of O_2 in DMSO, which has a half-wave potential of $-1.24\,V$ vs Fc^+/Fc. This means that O_2 can potentially react with reduced AQ via a redox process.

References

(S1) Bachman, J. E.; Curtiss, L. A.; Assary, R. S. Investigation of the Redox Chemistry of Anthraquinone Derivatives Using Density Functional Theory. *The Journal of Physical Chemistry A* **2014**, *118*, 8852–8860.

(S2) Glendening, E. D.; Landis, C. R.; Weinhold, F. NBO 6.0: Natural bond orbital analysis program. *Journal of Computational Chemistry* **2013**, *34*, 1429–1437.

(S3) Becke, A. D. Density functional thermochemistry. III. The role of exact exchange. *The Journal of Chemical Physics* **1993**, *98*, 5648–5652.

(S4) Lee, C.; Yang, W.; Parr, R. G. Development of the Colle-Salvetti correlation-energy formula into a functional of the electron density. *Phys. Rev. B* **1988**, *37*, 785–789.

(S5) Clark, T.; Chandrasekhar, J.; Spitznagel, G. W.; Schleyer, P. V. R. Efficient diffuse function-augmented basis sets for anion calculations. III. The 3-21+G basis set for first-row elements, Li-F. *J. Comput. Chem.* **1983**, *4*, 294–301.

(S6) Krishnan, R.; Binkley, J. S.; Seeger, R.; Pople, J. A. Self-consistent molecular orbital methods. XX. A basis set for correlated wave functions. *J. Chem. Phys.* **1980**, *72*, 650–654.

(S7) Marenich, A. V.; Cramer, C. J.; Truhlar, D. G. Universal Solvation Model Based on Solute Electron Density and on a Continuum Model of the Solvent Defined by the Bulk Dielectric Constant and Atomic Surface Tensions. *The Journal of Physical Chemistry B* **2009**, *113*, 6378–6396.
